# Estimating the divergence point: a novel distributional analysis procedure for determining the onset of the influence of experimental variables

**DOI:** 10.3389/fpsyg.2014.01432

**Published:** 2014-12-08

**Authors:** Eyal M. Reingold, Heather Sheridan

**Affiliations:** ^1^Department of Psychology, University of Toronto at MississaugaMississauga, ON, Canada; ^2^Centre for Vision and Cognition, Department of Psychology, University of SouthamptonSouthampton, UK

**Keywords:** fixation duration, reaction time, distributional analysis, divergence point analysis, individual differences, direct cognitive control, survival analysis

## Abstract

The divergence point analysis procedure is aimed at obtaining an estimate of the onset of the influence of an experimental variable on response latencies (e.g., fixation duration, reaction time). The procedure involves generating survival curves for two conditions, and using a bootstrapping technique to estimate the timing of the earliest discernible divergence between curves. In the present paper, several key extensions for this procedure were proposed and evaluated by conducting simulations and by reanalyzing data from previous studies. Our findings indicate that the modified versions of the procedure performed substantially better than the original procedure under conditions of low experimental power. Furthermore, unlike the original procedure, the modified procedures provided divergence point estimates for individual participants and permitted testing the significance of the difference between estimates across conditions. The advantages of the modified procedures are illustrated, the theoretical and methodological implications are discussed, and promising future directions are outlined.

Since the pioneering investigation of reaction time (RT) performance by Franciscus Donders (1868), the study of the time-course and speed of mental processes, which is often referred to as mental chronometry, constitutes an ongoing focus for research on perception, cognition and cognitive neuroscience. In particular, extensive research has been directed at examining variables that influence fixation duration and RT data. The latency with which a variable impacts performance on perceptual and cognitive tasks is the focus of much empirical work and theorizing in many domains of research. Much of this work has focused on the analysis of mean durations, but there is a growing recognition that distributional analyses can provide more fine-grained time-course information than mean analyses alone (e.g., Ratcliff and Murdock, 1976; Ratcliff, 1979; Heathcote et al., 1991; McConkie et al., 1994; McConkie and Dyre, 2000; Yang and McConkie, 2001; Feng, 2009; Staub et al., 2010; Balota and Yap, 2011; Staub, 2011; White and Staub, 2012; Luke and Henderson, 2013; Luke et al., 2013; Staub and Benatar, 2013).

Recently, Reingold et al. (2012) introduced a novel distributional analysis method aimed at determining the earliest discernible impact of a variable by contrasting survival curves across two experimental conditions and using a bootstrap resampling procedure (Efron and Tibshirani, 1994) for determining the point at which the two curves begin to diverge. To date, this *Divergence Point Analysis* (*DPA*) procedure has proven useful for obtaining fine-grained time-course information about the earliest impact of variables on fixation duration in a variety of domains including reading (Reingold et al., 2012; Sheridan and Reingold, 2012a,b; Sheridan et al., 2013; Glaholt et al., 2014; Inhoff and Radach, 2014; Schad et al., 2014), visual search (Reingold and Glaholt, 2014) and scene perception (Glaholt and Reingold, 2012). In addition, a recent study employed this technique to analyze the distributions of RT data that were obtained during the performance of a word recognition task (Ando et al., 2014).

The main goal of the present paper was to propose and evaluate two modified versions of the DPA procedure. The first modified procedure was aimed at the computation of confidence intervals for divergence point estimates in order to determine whether or not these estimates are significantly different across experimental conditions, while the second modified procedure was designed for computing divergence point estimates for individual participants. Accordingly, we begin by describing the technique used in prior studies and its limitation. Next we introduce the two modified DPA procedures. We then report on the findings from two simulations which were conducted in order to examine the robustness and consistency of the original and modified procedures. Finally, the performance of the modified procedures was examined by reanalyzing data that was obtained in several prior studies.

## THE DPA PROCEDURE – REINGOLD ET AL. (2012)

The DPA procedure was developed in order to test the hypothesis that cognitive influences could be rapid enough to produce an immediate adjustment of fixation duration based on the properties of the fixated stimulus (henceforth, *the direct cognitive control hypothesis*). Prior to the 1970s, there was a great deal of skepticism over whether cognitive processes could have a rapid impact on fixation duration. This was in part based on the widely held belief that cognition was simply too slow to produce real-time adjustment of eye-movement parameters (e.g., Kolers, 1976). Although, extensive research during the decades to follow produced ample evidence for cognitive influences on mean fixation duration, the direct cognitive control hypothesis remained controversial (for reviews see Rayner, 1998, 2009; Reingold et al., 2012, in press). This is because an influence of a cognitive variable on mean fixation duration might be restricted to very few long fixations while the vast majority of fixations remain unaffected. In order to address this issue, Reingold et al. (2012) argued that an analysis of the distribution of fixation durations has the potential to provide a more direct estimate for the earliest discernible influence of a variable on fixation duration (see Staub et al., 2010 for a similar argument).

**FIGURE 1:**
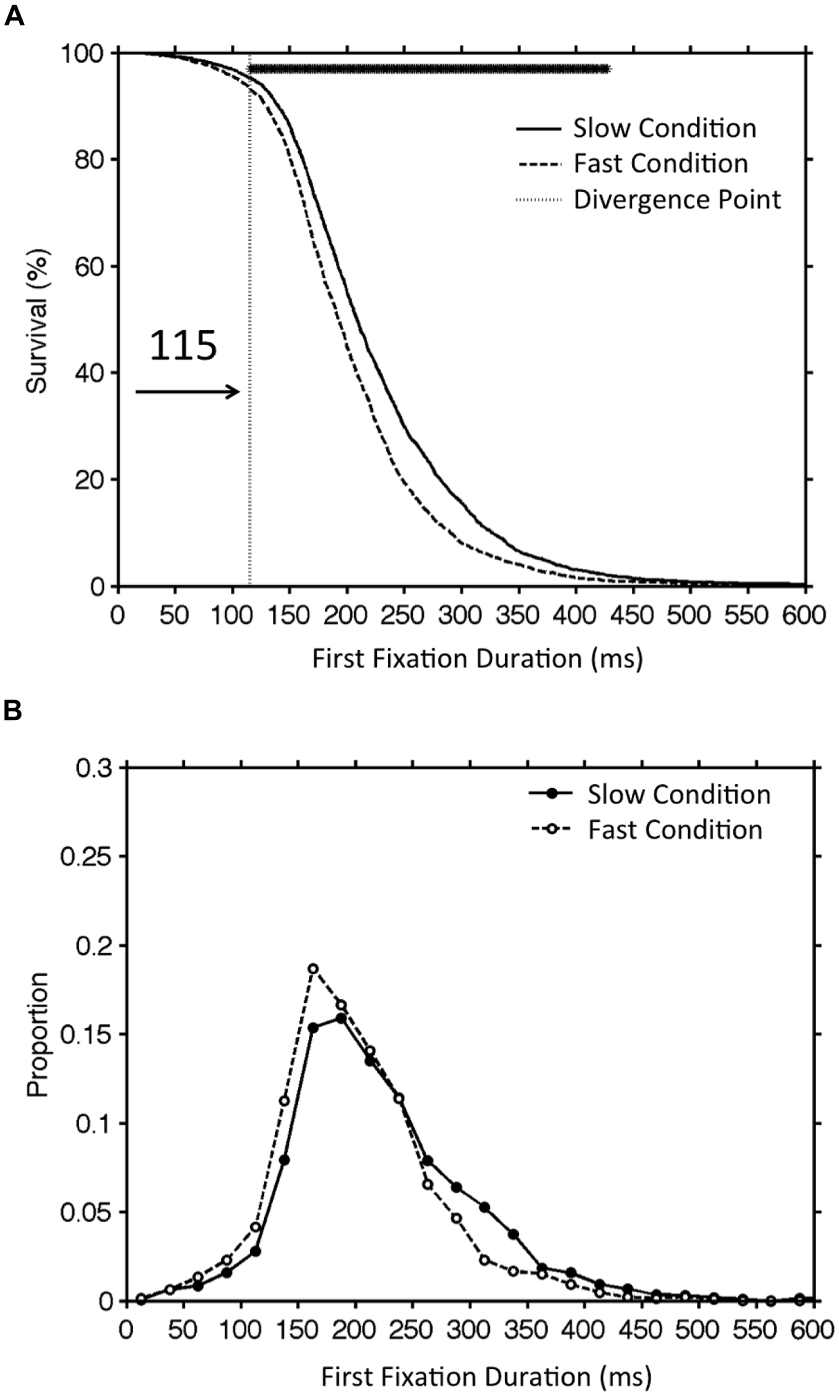
**An illustration of the original divergence point analysis (DPA) procedure.** Panel **(A)** shows the survival curves for first fixation duration in the slow and fast conditions. The vertical line marks the divergence point estimate and the row of asterisks at the top of this panel indicates 1-ms time bins for which the survival percent was significantly greater for the slow than the fast condition. Panel **(B)** provides the corresponding histograms of fixation duration.

To illustrate the DPA procedure, **Figures 1A,B** displays the survival curve and the histogram of fixation duration for two experimental conditions that differ in terms of the mean fixation duration (henceforth referred to as the fast vs. slow condition). To plot survival curves, the *survival percent* for a given time *t*, refers to the percent of fixations with a duration greater than *t* (i.e., “surviving” fixation are those that were not yet terminated by a saccade). As can be seen in **Figure 1B**, survival is at one hundred percent when *t* equals zero (since all of the fixation durations by definition are greater than zero). Survival percent then declines as *t* increases and approaches zero percent as *t* approaches the duration of the longest observed fixation duration. Thus, the survival curve depicts a monotonically decreasing function with an initial slow decrease that is followed by a rapid, largely linear decrease in the middle of the curve, and with a final section of slow decline. The shape of the survival curve reflects the outline of the histogram of fixation duration which is approximately normal yet presents a clear rightward (positive) skew (see **Figure 1B**). In other words, there are more fixations near the center of the distribution than on either tail, and the right tail of the distribution is more densely populated than the left tail.

To pinpoint the earliest significant effect of a variable, the DPA procedure uses a bootstrap resampling technique (Efron and Tibshirani, 1994; for an extensive recent review and bibliography see Chernick, 2008). Bootstrapping is a non-parametric approach to statistical inference. In traditional parametric inference, statistics computed for a given sample are used to make inferences about the population parameters on the basis of *a priori* assumptions and analytic formulas that fully specify the sampling distribution of these statistics. In marked contrast, the bootstrapping approach does not rely on such strong distributional assumptions and/or on the availability of analytic formulas. Instead, the bootstrapping procedure involves approaching the sample as if it was the population and using repeated, multiple iterations of random resampling with replacement (sometime referred to as Monte Carlo sampling) to empirically derive an estimate of the entire sampling distribution of a statistic that is computed on every iteration. Thus, to the extent that the sample is in fact representative of the population, the bootstrap resampling technique offers a very powerful and flexible tool for making statistical inferences under conditions in which the traditional parametric approach offers no solutions.

Employing such an approach, Reingold et al. (2012) used 10,000 iterations of random resampling of fixations for each participant and condition. For each iteration of the bootstrap procedure, survival curves were generated for each individual participant. Next, for each 1-ms bin ranging from 1 to 600 ms, survival percent values were averaged across participants to produce the group survival curves. Using the group survival curves, for each bin, the value in the fast condition was then subtracted from the corresponding value in the slow condition. Across the 10,000 iterations, the obtained differences for each bin were then sorted in order of magnitude and the range between the 5th and the 9,995th value was defined as the confidence interval of the difference for each bin. Time bins for which the lower bound of the confidence interval of the difference between the slow and fast survival curves was greater than zero were considered to represent a significant difference between curves. The divergence point estimate was then defined as the earliest significant difference point that was part of a run of five consecutive significant difference points (For an illustration of significant bins and divergence point estimate see **Figure 1A**; See supplementary materials for Matlab code implementation of this procedure).

It is important to note, that Reingold et al. (2012) and follow-up investigations (e.g., Glaholt and Reingold, 2012; Sheridan and Reingold, 2012a,b; Sheridan et al., 2013; Glaholt et al., 2014; Reingold and Glaholt, 2014) attempted to test the validity of the direct cognitive control hypothesis that predicted early divergence points as a function of cognitive influences. Consequently, in order to protect against making a Type I error (i.e., erroneously estimating a divergence point prior to the actual point of divergence), the DPA procedure incorporated very conservative criteria for estimating divergence points (i.e., α < 0.001 for the significance of individual bins and the requirement for five consecutive significant bins). Importantly, despite this deliberate bias against the direct cognitive control hypothesis, the above studies documented fast acting cognitive influences in strong support for that hypothesis (for a review see Reingold et al., in press). However, the cost of such a conservative bias in the DPA procedure is the risk that the estimate of the divergence point would be delayed relative to the actual point of divergence. This would be especially the case under low experimental power (i.e., a small number of participants and observations). To mitigate this risk, the above investigations employed a large number of observations and participants. Nevertheless, it would be desirable to construct a version of the DPA technique that can successfully handle the lower experimental power that is typical of many eye movements and reaction time experiments.

Another limitation of the current version of the DPA procedure is that it does not provide confidence intervals for the obtained divergence point estimates and consequently it does not permit determining whether or not estimates of divergence points are significantly different across experimental conditions. Finally, the analysis method used in prior studies derived divergence point estimates based on group data rather than for each individual participant. While computing divergence point estimates for each participant might prove useful in the context of individual differences research, a key challenge for this endeavor concerns the small number observations that are typically available for an individual participant in each condition. Yet, despite this inherent low power, recent findings suggest that distributional analysis techniques could be successfully used to produce reliable individual differences measures which were shown to be correlated with measures of cognitive ability, such as working memory capacity (e.g., Schmiedek et al., 2007; Tse et al., 2010; Balota and Yap, 2011; Staub and Benatar, 2013). Next, we propose and evaluate the modified DPA procedures that attempted to address the limitations of the method that was used in prior studies.

## THE MODIFIED DPA PROCEDURES

In the present paper we propose two modified versions of the DPA procedure that was introduced by Reingold et al. (2012). Specifically, the *Confidence Interval DPA* procedure was aimed at the computation of confidence intervals for divergence point estimates in order to determine whether or not these estimates are significantly different across experimental conditions, while the *Individual Participant DPA* procedure was designed for computing divergence point estimates for each participant in the sample. In order to compare the performance of the original and modified DPA procedures, as a function of experimental power, we conducted two simulations. The first simulation (*Simulation 1*) examined the accuracy and variability of divergence point estimates that were obtained when a large sample of 104 participants was progressively reduced by randomly selecting subsets of 52, 26, or 13 participants. In the second simulation (*Simulation 2*) the datasets corresponding to the 104 participants from Simulation 1 were modified to create artificial datasets with known divergence point values which were used to investigate the number of observations required per condition in order to accurately estimate individual differences in divergence point values. Specifically, across participants, simulated divergence point values varied from 110 to 210 ms and the Individual Participant DPA procedure was used to produce divergence point estimates. The strength of the correlation between simulated and obtained divergence point estimates was evaluated when the number of observations for each participant was progressively reduced by randomly selecting subsets of 36, 24, or 12 fixations per condition. Finally, the performance of the original and modified procedures was examined by reanalyzing data that was obtained in several prior reading studies.

## METHOD

In this section we first describe the details of the modified DPA procedure (See supplementary materials for Matlab code implementations of these procedures). We then outline the method used in Simulations 1 and 2.

### CONFIDENCE INTERVAL DPA PROCEDURE

This procedure was identical to the method used by Reingold et al. (2012) procedure with the following exceptions: (1) 1,000 rather than 10,000 bootstrap iterations were used, and (2) the divergence point was calculated for each iteration rather than once across all iterations. Specifically, in the Confidence Interval DPA procedure, for each iteration, the divergence point estimate was defined as the first 1-ms bin in a run of five consecutive bins in which the survival percent in the slow condition was at least 1.5% greater than the survival percent in the fast condition. Across the 1,000 iterations, divergence point estimates were then sorted from the smallest to the largest value and the 25th and 975th values constituted the 95% confidence interval. In addition, the median of the 1,000 divergence point values was used as the divergence point estimate for the sample. It is important to note that the threshold we selected for the detection of a difference between survival percent across the slow and fast conditions (1.5%) necessarily implies that a smaller effect would not be detected by the Confidence Interval DPA procedure. However, setting a lower threshold would risk falsely detecting a difference under noisy low power conditions. Thus, although this threshold can be safely lowered for very large samples, based on our explorations to date, we believe that the 1.5% difference threshold constitutes an optimal compromise under the typical experimental power that is used in the literature.

### INDIVIDUAL PARTICIPANT DPA PROCEDURE

As explained earlier, accurately estimating the divergence point for each individual participant is far from trivial given the small number observations that are typically available for each condition. Consequently, we designed the Individual Participant DPA procedure to maximize the chances of correctly detecting true divergence while minimizing the chances of falsely detecting divergence due to noisy individual participant’s survival curves. Specifically, in order to derive the individual divergence estimates, the following sequence of steps was performed separately for the fixation duration data from each participant. For each of 1,000 bootstrap iterations, regardless of the number of fixations that were available for a given participant, 1200 fixations were randomly sampled with replacement from the pool of fixations corresponding to the slow condition and from the pool of fixations corresponding to the fast condition. Both sets of 1200 fixations were sorted from the shortest to the longest duration value and then paired (i.e., *s_1_ f_1_, s_2_ f_2_, s_3_ f_3_,…,s_1200_ f_1200_*). For both the slow and fast condition, the sorted order of fixations specified the sequence in which fixations were terminated, and in turn the “death” of a single fixation decreased the survival percent by the minimum possible decrement (i.e., *1/1200^∗^100*). Consequently, the process of sorting by fixation duration created 1200 survival percent bins (for bin *i* survival percent equalled *100-i/1200^∗^100)*. For each of the 1200 survival percent bins, the difference between the duration of the slow minus the fast fixation duration was computed (for bin *i,* this difference equalled *s_i_ – f_i_)*. Next, we identified the first survival percent bin in a run of 100 consecutive bins in which the value of this difference was positive (*s_i_* > *f_i_*). Finally, the average duration of the pair of fixations corresponding to that bin was defined as the divergence point value for each iteration. Iterations in which a divergence point value was not identified were discarded and the median value across the remaining iterations was then defined as the divergence point estimate for that individual. Participants for which a divergence point value was obtained in less than 50% of iterations were deemed unreliable and were excluded from the computation of group divergence point estimates (the frequency with which this occurs was recorded). It is important to note that the threshold we selected for the detection of a difference between the slow and fast survival curves in each iteration (i.e., 100 consecutive bins in which s_i_ > f_i_) necessarily implies that a weak and/or brief true divergence effect might be missed at least on some iterations. However, given the small number observations that are typically available for each participant, a more lenient detection criterion would inevitably result in an unacceptably high false alarm rate. Thus, although the above detection criterion can be made more lenient in the context of studies with large numbers of observations for each participant and condition, based on our explorations to date, we believe that it constitutes an optimal compromise under the typical experimental power that is used in the literature (see Simulation 2 for a related investigation).

### SIMULATION 1

For the purpose of the illustration and evaluation of the modified DPA procedures, we used a large dataset obtained by Sheridan et al. (2013; Experimental 1A, normal reading condition) in which the word frequency of target words was manipulated (low frequency vs. high frequency) and the distribution of first-fixation durations on target words was contrasted across conditions. This dataset was composed of 104 participants with approximately 60 observations per condition for each participant. We simulated a reduction in experimental power by treating the dataset of 104 participants as the population and randomly sampling without replacement subsets of 52, 26, or 13 participants. For each sample size, 100 random samples were drawn and estimates of divergence points were computed based on both the original and modified DPA procedures.

### SIMULATION 2

To produce the artificial datasets that were used in this simulation we modified the datasets corresponding to the 104 participants from Simulation 1. Specifically, the sample of 104 participants was randomly divided into 26 groups of four participants. Each group was then assigned a unique simulated divergence point value that varied between 110 and 210 ms (i.e., increasing across groups by 4 ms steps: 110 ms, 114 ms, 118 ms,....,210 ms). To implement these simulated divergence points, the fast condition data for each participant in Simulation 2 was identical to the data used in Simulation 1 (i.e., the pool of fixations on high frequency target words from Sheridan et al., 2013). In contrast, the slow condition was created by modifying the fast condition data rather than by using the empirical data. Importantly, the slow condition in the artificial datasets was constructed as follows: (1) all fixations with durations that were shorter than the simulated divergence point value were identical across the fast and slow conditions, and (2) a randomly selected subset of 50% of fixations in the fast condition with durations that were longer than or equal to the simulated divergence point value were lengthened by 50 ms to create their counterparts in the slow condition (the remaining 50% of longer fixations were identical across the fast and slow conditions). The main goal of Simulation 2 was to examine the strength of the correlation between simulated divergence points and the estimates generated by the Individual Participant DPA procedure. This correlation indicates the extent to which individual differences are accurately depicted by the estimates derived from Individual Participant DPA procedure. In addition to computing this correlation using the 104 artificial datasets that were described above, it was also computed when these datasets were progressively reduced by randomly (without replacement) and repeatedly selecting subsets of 36, 24, or 12 fixations per condition (100 random samples of 36, 24, or 12 fixations per condition were produced).

## RESULTS

In this section we first report on the results of Simulations 1 and 2 and then the performance of the original and modified procedures were examined by reanalyzing data that was obtained in several prior reading studies.

### SIMULATION 1

**FIGURE 2:**
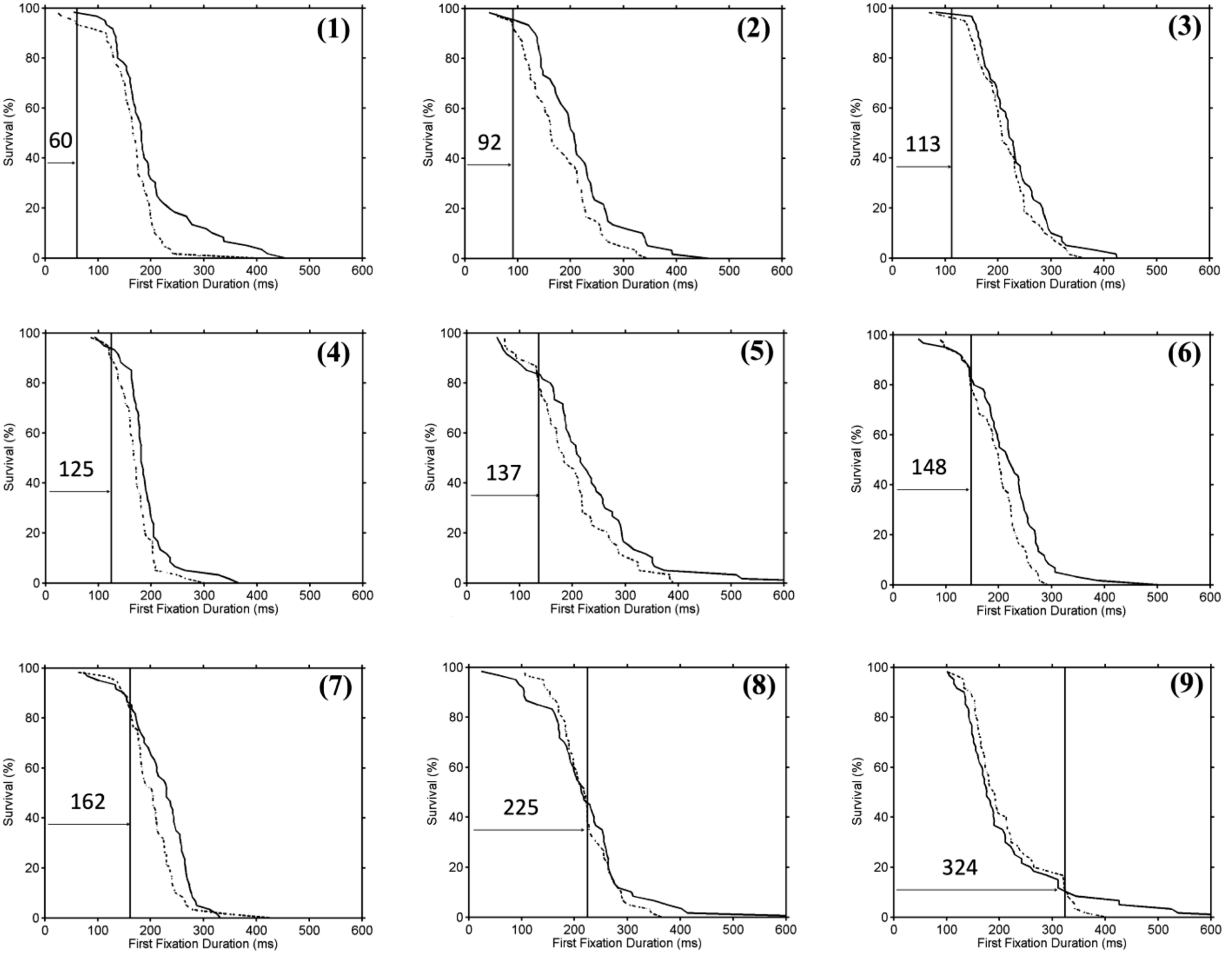
**An illustration of the range of divergence point estimates that were obtained across individual participants from the sample of 104 participants which was used for Simulation 1.** Survival curves (slow condition = solid line, fast condition = dashed line) for 9 participants with progressively longer divergence point estimates are shown in Panel **(1–9)**. The vertical line in each panel marks the divergence point estimate (see text for details).

For the full sample of 104 participants, we calculated divergence point estimates for all 3 versions of the DPA procedure. For the group data, the analysis using the original DPA procedure and the Confidence Interval DPA procedure yielded nearly identical divergence point estimates (original DPA = 112 ms; Confidence Interval DPA = 114 ms). Importantly, the Confidence Interval DPA procedure also provided a 95% confidence interval (98–130 ms). In contrast, the Individual Participant DPA procedure produced an average divergence point estimate across individual participants (*M* = 131 ms; SD = 51.1) that was somewhat longer than the divergence point estimates that were produced using the group data. This is the case because the DPA procedures that are based on group data detect a divergence at the point at which participants in the sample with early divergence point estimates begin to significantly influence the group survival curves. Thus, the Confidence Interval DPA and the Individual Participant DPA procedures should be considered supplementary as they contribute unique information about the characteristics of the point of divergence across the sample. **Figure 2** illustrates the wide spectrum of divergence point estimates that was observed across the sample of 104 participants. Not surprisingly, survival curves for individual participants were fairly noisy. However, a visual inspection of **Figure 2** indicates that the point of divergence identified by the Individual Participant DPA procedure appears quite reasonable.

**FIGURE 3:**
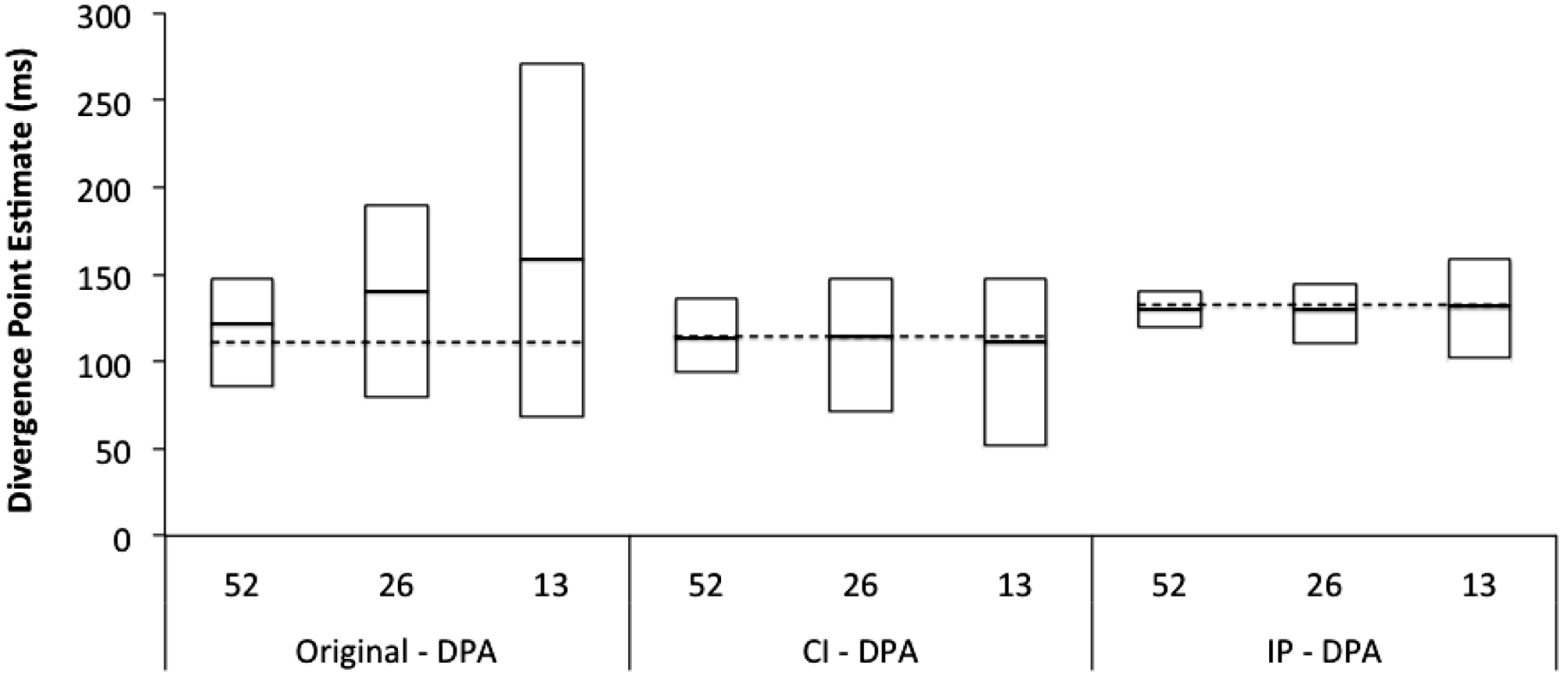
**The results from Simulation 1 that examined the accuracy and variability of divergence point estimates as a function of sample size (52, 26, 13) and DPA procedure (CI-DPA, Confidence Interval DPA procedure; IP-DPA, Individual Participant DPA procedure).** For each condition, the minimum, maximum and average values of the estimates are displayed. The dashed horizontal lines show the criterion divergence estimates that were obtained by using each version of the DPA procedure to analyze data from the entire sample of 104 participants (see text for details).

The results of Simulation 1 provided a more systematic method for evaluating the performance of the original and modified DPA procedures. Specifically, as shown in **Figure 3**, for each of the 3 versions of the DPA procedure (original, Confidence Interval DPA, Individual Participant DPA), we used the divergence point estimates from the sample of 104 participants as the criterion for evaluating the estimates that were produced when a random subset of the large sample was used for computing divergence point estimates. Next, for each sample size (52, 26, and 13), 100 random samples without replacement were drawn, and estimates of divergence points were computed based on all 3 versions of the DPA procedure. **Figure 3** shows the minimum, maximum and average values of the divergence point estimate by sample size and procedure. The results of the simulation indicated that as sample size decreased the modified procedures performed substantially better than the original procedure. As expected, when sample size decreased the original DPA procedure produced estimates that were biased to be longer than the criterion divergence value (i.e., the divergence value in the full sample of 104 participants). While this bias was relatively small when the sample size was cut in half, it became much more pronounced for smaller sample sizes. In marked contrast, both of the modified DPA procedures provided an unbiased estimate of the criterion divergence point regardless of sample size. In addition, the variability of the divergence point estimates across the randomly sampled subsets was substantially smaller in the modified procedures than the original procedure (see **Figure 3**). As a case in point, for a sample size of 52, the Confidence Interval DPA procedure produced a range of divergence point estimates of 94–136 ms, and this range is almost as small as the 95% confidence interval which was obtained using the Confidence Interval DPA procedure on the full sample of 104 participants.

### SIMULATION 2

**FIGURE 4:**
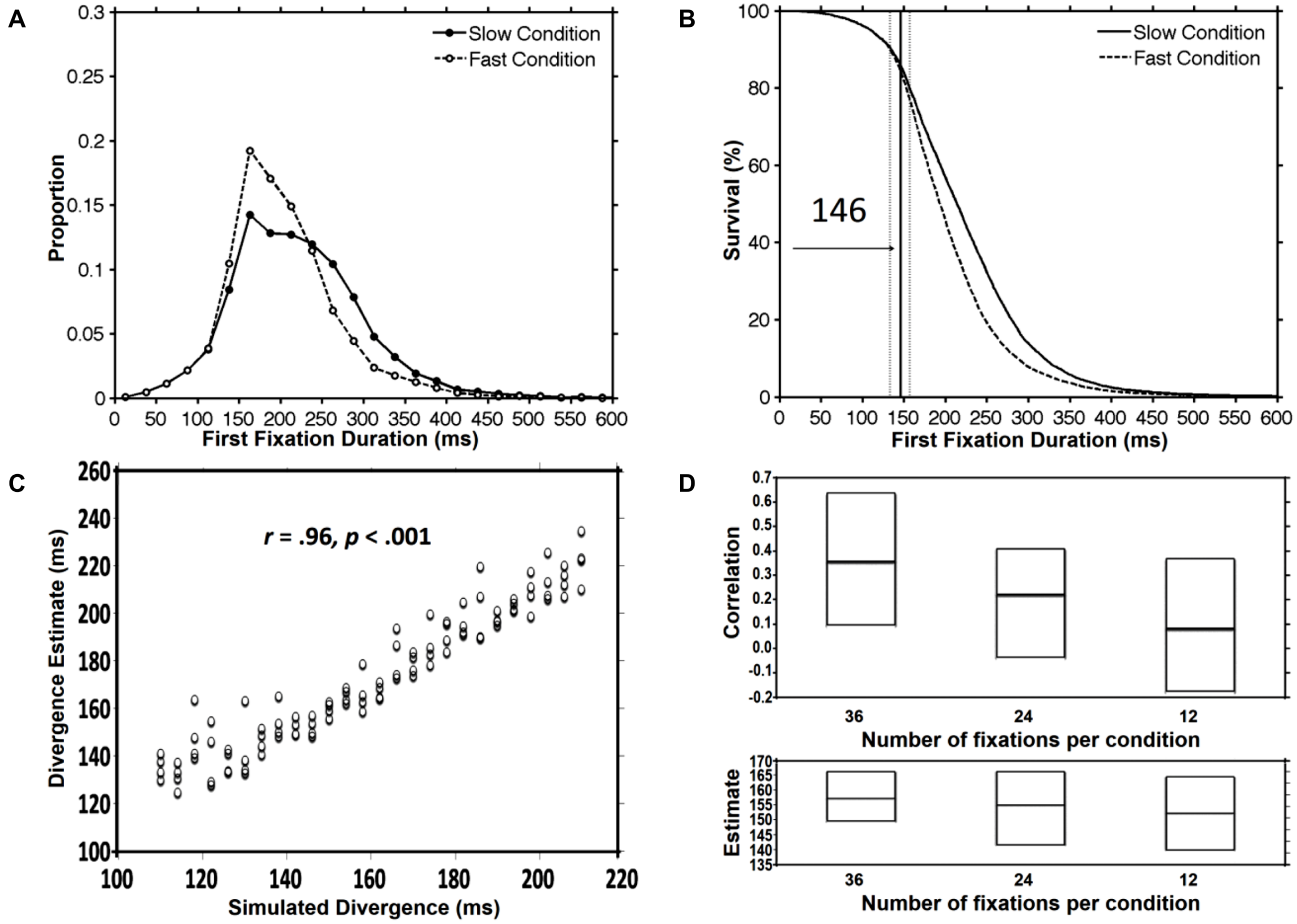
**The results from Simulation 2 that used artificial datasets with known divergence point values which varied between 110 and 210 ms (*M* = 160 ms).** Panel **(A)** shows histograms of the slow and fast condition across the sample of 104 participants. Panel **(B)** shows the divergence point estimate (solid line) and the 95% confidence interval (dotted lines) that were produced using the Confidence Interval DPA procedure. Panel **(C)** shows a scattergram with each dot representing the simulated and obtained divergence point for an individual participant (using the Individual Participant DPA procedure). Panel **(D)** shows, across 100 randomly selected subsets of 36, 24, or 12 fixations per condition, the minimum, maximum, and average values of (1) the correlation between simulated and obtained divergence, and (2) mean divergence point estimate across participants (see text for details).

As explained earlier, for the purpose of this simulation, we created artificial datasets corresponding to 104 participants with simulated divergence points that varied between 110 and 210 ms. **Figure 4A** displays the histograms of fixation duration for the fast condition (*M* = 203 ms; SD = 26.7) and the slow condition (*M* = 221 ms; SD = 29.7). We began by calculating divergence point estimates using all 3 versions of the DPA procedure. All procedures yielded estimates that were fairly close to the average simulated divergence point across participants which was 160 ms. However, as was the case for Simulation 1, the Individual Participant DPA procedure produced an average divergence point estimate across individual participants (*M* = 173 ms; SD = 28.6) that was somewhat longer than the divergence point estimates that were produced using the group data (original DPA = 152 ms; Confidence Interval DPA = 146 ms; see **Figure 4B**). Most importantly, as shown in **Figure 4C**, the Individual Participant DPA procedure was very accurate in detecting the simulated divergence points as reflected by a very strong correlation between simulated and obtained divergence points across the sample of 104 participants [*r*(102) = 0.96, *p* < 0.001].

The main goal of Simulation 2 was to examine the variation in the strength of this correlation as a function of the number of available observations per condition. Specifically, for each participant, the number of fixations was progressively reduced by randomly and repeatedly selecting subsets of 36, 24, or 12 fixations per condition. For each subset size, 100 reduced datasets were produced and the correlation between simulated and obtained divergence points was computed across the sample of 104 participants. **Figure 4D** shows the minimum, maximum and average values of the correlation by subset size (36, 24, or 12). As can be seen in this figure, a decrease in the number of fixations per condition, from a maximum of 60 in the unreduced datasets to 36, produced a dramatic decrease in the average correlation between simulated and obtained divergence points (0.96–0.36). This correlation was further reduced for a subset size of 24 fixations per condition (*r* = 0.22) and was all but eliminated for a subset size of 12 fixations per condition (*r* = 0.08). Interestingly, as shown in the figure, despite this very substantial decrease in the magnitude of the correlation, the average divergence point estimate across participants remained relatively stable and accurate (subset size 36 = 157; subset size 24 = 155; subset size 12 = 152). This pattern of findings clearly indicates the need for a sufficient number of observations per condition in studies which aim to use divergence point estimates in individual differences research. However, the Individual Participant DPA procedure might still be useful as a measure of the average divergence point estimate even with a much smaller number of observations per condition.

### RE-ANALYSIS OF DATA FROM PRIOR STUDIES

The findings from several prior studies were analyzed using the original and modified DPA procedures and the obtained estimates are summarized in **Table 1**. As can be seen by an inspection of the table, the estimates produced by the modified procedures are generally very consistent with the published estimates that were based on the original DPA procedure. This was especially the case for experimental variables (e.g., word frequency, preview validity, and location) which produced strong effects on fixation duration (i.e., effects with a large mean fixation duration difference between the slow and fast conditions) and/or very consistent effects (i.e., effects for which the Confidence Interval DPA procedure produced a narrow confidence interval). In contrast, for variables which produced effects that were smaller and more variable (e.g., predictability, and lexical ambiguity), the Confidence Interval DPA produced somewhat earlier divergence estimates than either the original or the Individual Participant DPA procedures. Additional studies are required in order to more fully explore the influence of effect size and consistency on divergence point estimates.

**Table 1:** Summary of the re-analysis of data from prior reading studies by DPA procedure.

		Mean duration (ms)			DPA procedure		
Variable	Study	Slow	Fast	Difference	Original	CI-DPA	IP-DPA
Word frequency (low–high frequency)	Reingold et al. (2012) valid preview	234	214	20	145	138 (131–147)	139 (59/60)
Predictability (low–high predictability)	Sheridan and Reingold (2012a)	216	208	8	140	124 (101–142)	138 (55/60)
Lexical ambiguity (subordinate–dominant context)	Sheridan and Reingold (2012b)	228	216	12	139	121 (96–150)	131 (53/60)
Preview validity (invalid–valid)	Reingold et al. (2012)	256	224	32	132	135 (129–147)	133 (59/60)
Location (central–outer location)	Reingold et al. (2012) valid preview	228	214	14	145	139 (131–148)	140 (58/60)

*DPA, Divergence point analysis; for mean fixation duration Difference, slow minus fast fixation duration; CI-DPA, Confidence Interval DPA procedure; IP-DPA, Individual Participant DPA procedure; for the Confidence Interval DPA procedure, the confidence interval is shown in brackets beside the estimate; for the Individual Participant DPA procedure, the number of participants out of 60 for which a valid estimate was produced is shown in brackets beside the estimate.*

**FIGURE 5:**
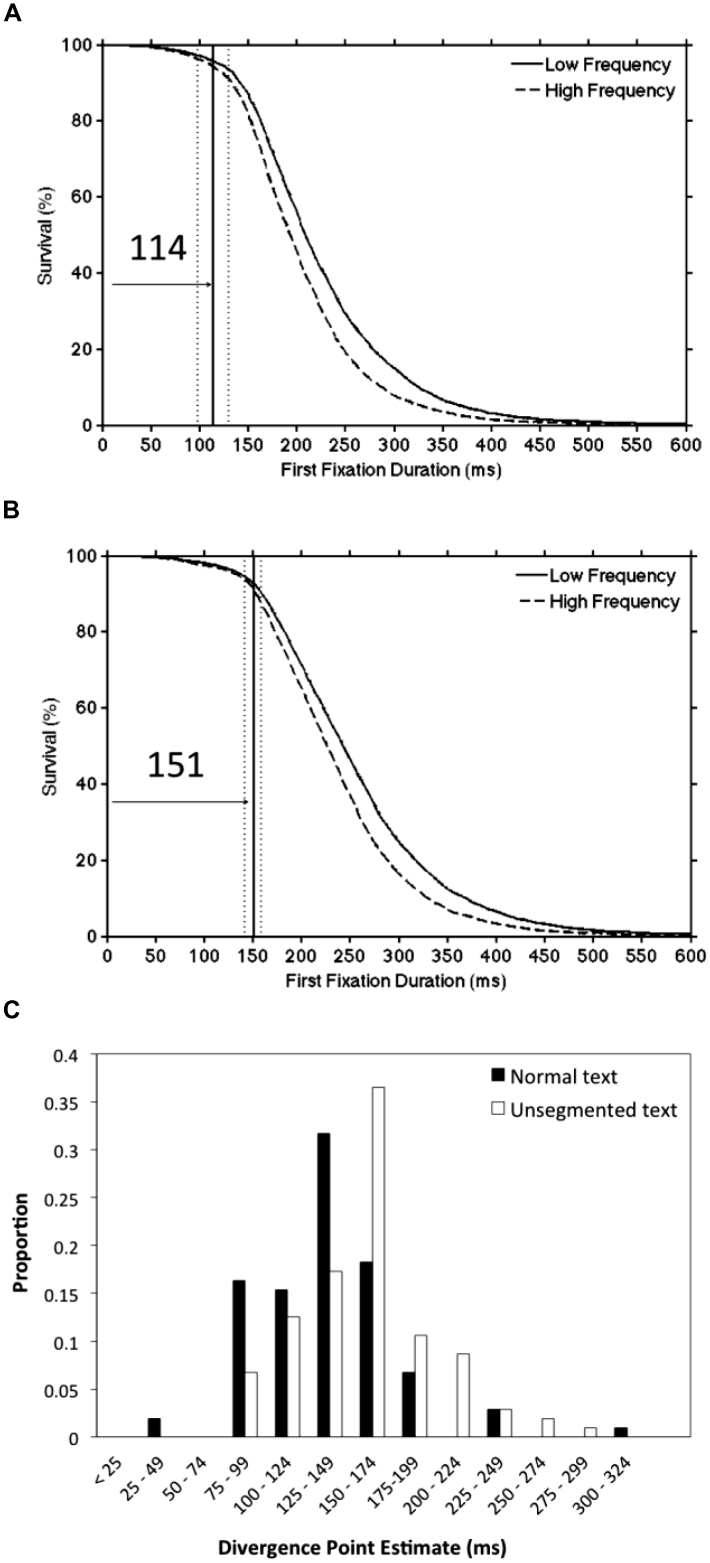
**A re-analysis of data from Sheridan et al. (2013) using the modified DPA procedures.** Panel **(A)** shows the word frequency divergence point estimate (solid line) and the 95% confidence interval (dotted lines) for the normal (segmented) text condition. Panel **(B)** presents the corresponding values for the unsegmented text condition. Panel **(C)** displays the distribution of divergence point estimates for individual participants as a function of text segmentation condition (see text for details).

To further evaluate DPA procedures, it is important to consider studies which involve comparing the time-course of the influence of variables under different experimental conditions. For example, Sheridan et al. (2013) investigated the hypothesis that lexical processing would be delayed by the removal of inter-word spaces (see Rayner et al., 1998). To do this, Sheridan et al. (2013) conducted an experiment in which high- and low-frequency words were embedded either in normal (English) text or in unsegmented text in which the blank spaces between the words were replaced with random numbers. Sheridan et al. (2013) demonstrated that the word frequency divergence points were delayed (by 23–40 ms) in the unsegmented condition relative to the normal reading condition. To examine whether this difference was in fact significant, we used the modified DPA procedures and reanalyzed the data obtained by Sheridan et al. (2013, Experiment 1A). The word frequency divergence point estimate reported by Sheridan et al. (2013) using the original DPA procedure was 112 ms in the normal (segmented) text condition and 152 ms in the unsegmented text condition. As shown in **Figures 5A,B**, the divergence point estimates that were obtained using the Confidence Interval DPA procedure (segmented = 114 ms; unsegmented = 151 ms) were extremely similar to the published estimates. More importantly, the 95% confidence intervals did not overlap between these conditions (segmented = 98 to 130 ms; unsegmented = 141 to 158 ms) indicating that the difference in divergence points as a function of segmentation was significant. A similar pattern of findings was obtained using the Individual Participant DPA procedure. Specifically, the average divergence point estimates were 131 ms in the segmented text condition and 155 ms in the unsegmented text condition and this difference was significant [*t*(103) = 3.63, *p* < 0.001]. In addition, **Figure 5C** shows the distribution of divergence point estimates for individual participants by text segmentation condition, and an inspection of this panel demonstrates a clear rightward shift in the unsegmented distribution as compared to the normal (segmented) text condition.

Taken together, the results of the simulations and re-analysis indicate that the original and modified DPA procedures performed similarly with a large sample size, but with reduced experimental power the modified procedures performed substantially better. In addition, the modified procedures allowed for testing the significance of the difference between divergence point estimates across experimental conditions. Finally, the Individual Participant DPA procedure has a distinct advantage over the DPA procedures that are based on group data. This is the case because this procedure has the potential to provide a much more complete and accurate description of the entire distribution of divergence point estimates across the sample. Furthermore, deriving divergence point estimates for each individual participant would potentially offer an interesting correlate for use in individual differences research. However, as shown by Simulation 2, a prerequisite for such an endeavor would be the availability of a sufficient number of observations per participant. In contrast, the Confidence Interval DPA procedure might be advantageous when exploring weak and inconsistent empirical effects or when the number of available observations per participant is relatively small.

## DISCUSSION

The goal of the present paper was to further develop the DPA procedure which was introduced by Reingold et al. (2012) as a method for investigating the direct cognitive control hypothesis. The basic experimental strategy which guided previous studies using this procedure was to employ large sample sizes and large numbers of observations per condition. In addition, given the persistent skepticism concerning the feasibility of the direct cognitive control hypothesis, the procedure incorporated a conservative criterion that made it more difficult to obtain evidence supporting this hypothesis. Despite this built in bias, the findings that emerged from this paradigm provided strong support for the validity of the direct cognitive control hypothesis (e.g., Glaholt and Reingold, 2012; Reingold et al., 2012; Sheridan and Reingold, 2012a,b; Sheridan et al., 2013; Glaholt et al., 2014; Inhoff and Radach, 2014; Reingold and Glaholt, 2014; Schad et al., 2014).

Although the original DPA procedure offered a useful tool for the investigation of the theoretical question which motivated its inception, further extensions of this technique seemed necessary before the DPA method could be applied more broadly across a wide range of experimental paradigms. The goal of the present manuscript was to augment the DPA procedure in several important ways: (1) by developing a DPA procedure that could provide confidence intervals for divergence point estimates, (2) by developing a DPA procedure that would be less negatively impacted by lower experimental power (although a large pool of observations is still the recommended approach for distributional analyses), and (3) by developing a method for obtaining divergence point estimates for individual participants.

Based on the present results, we argue that the modified DPA procedures constitute substantial progress toward attaining these goals. However, it should be acknowledged that the DPA procedure is still in its infancy. Much more methodological and empirical work is required in order to fully evaluate the merit and the scope of this technique. Future directions might include attempting to develop an analytic method for producing divergence point estimates that could then be compared with the non-parametric bootstrapping approach (Kullback and Leibler, 1951; Matuschek and Risse, in preparation). It would also be interesting to investigate the value of the divergence point estimate as a predictor variable in individual differences research. Another potentially promising future direction would be to extend the use of the procedure to investigate different types of response latencies. To date, there was only one study that used the DPA procedure to analyze RT data (Ando et al., 2014), and all of the other studies examined fixation duration data. In principle, the DPA procedure could accommodate a variety of behavioral and/or neural response indicators such as reaction time, fixation duration, or changes in electrophysiological measures.

For all types of response modalities, one important issue to consider is that there is necessarily a delay between the point at which the cognitive system first differentiates between two categories of stimulus states (e.g., familiar vs. unfamiliar stimuli), and the point at which significant differential responding is detected. Inferring the time-course of cognitive and neural processes is a complex enterprise that requires taking into account various neural transmission delays and response output delays. In addition, the detection of differential responding by measurement and statistical instruments requires a minimum intensity and/or duration to occur. This issue inevitably distorts time-course estimates, and the magnitude of the inaccuracy can be substantial for weak effects or effects that have a very gradual onset. Given that no single procedure or paradigm is capable of providing artifact-free and assumption-free time-course estimates, our approach has been to seek convergent evidence by employing multiple distributional analysis methods (e.g., survival analysis and Ex-Gaussian fitting), and by comparing our findings to those obtained from neuroimaging studies using ERP and MEG methodology (for a review see Reichle and Reingold, 2013).

Finally, with respect to the direct cognitive control hypothesis, we would suggest that the focus in the field is beginning to shift away from an “existence proof” stage (i.e., the search for a convincing demonstration of immediate cognitive influences on eye movement parameters). Instead, there seem to be greater focus on deriving quantitative estimates for the timing and magnitude of the influence of cognitive variables. Such estimates constitute critical “benchmarks” for the development and testing of computational models of eye-movement control in visual cognition. We would strongly argue that the DPA procedure has the potential to contribute to this important goal.

## Conflict of Interest Statement

The authors declare that the research was conducted in the absence of any commercial or financial relationships that could be construed as a potential conflict of interest.
